# Molecular characteristics and therapeutic implications of Toll-like receptor signaling pathway in melanoma

**DOI:** 10.1038/s41598-023-38850-y

**Published:** 2023-09-04

**Authors:** Hewen Guan, Xu Chen, Jifeng Liu, Jiaao Sun, Hui Guo, Yuankuan Jiang, Huimin Zhang, Biao Zhang, Jingrong Lin, Qihang Yuan

**Affiliations:** 1 Department of Dermatology, First Affiliated Hospital of Dalian Medical University, Dalian, Liaoning China; 2 Department of General Surgery, First Affiliated Hospital of Dalian Medical University, Dalian, Liaoning China; 3 Department of Urology, First Affiliated Hospital of Dalian Medical University, Dalian, Liaoning China; 4 Department of Oncology, First Affiliated Hospital of Dalian Medical University, Dalian, China

**Keywords:** Computational biology and bioinformatics, Oncology, Pathogenesis

## Abstract

Melanoma is a malignant tumor of melanocytes and is often considered immunogenic cancer. Toll-like receptor-related genes are expressed differently in most types of cancer, depending on the immune microenvironment inside cancer, and the key function of Toll-like receptors (TLRs) for melanoma has not been fully elucidated. Based on multi-omics data from TCGA and GEO databases, we first performed pan-cancer analysis on TLR, including CNV, SNV, and mRNA changes in TLR-related genes in multiple human cancers, as well as patient prognosis characterization. Then, we divided melanoma patients into three subgroups (clusters 1, 2, and 3) according to the expression of the TLR pathway, and explored the correlation between TLR pathway and melanoma prognosis, immune infiltration, metabolic reprogramming, and oncogene expression characteristics. Finally, through univariate Cox regression analysis and LASSO algorithm, we selected six TLR-related genes to construct a survival prognostic model, divided melanoma patients into the training set, internal validation set 1, internal validation set 2, and external validation set for multiple validations, and discussed the correlation between model genes and clinical features of melanoma patients. In conclusion, we constructed a prognostic survival model based on TLR-related genes that precisely and independently demonstrated the potential to assess the prognosis and immune traits of melanoma patients, which is critical for patients’ survival.

## Introduction

Melanoma is a deadly type of human skin cancer that has increased over the past 40 years, rising faster than any other solid tumor, and has a high metastasis and fatality rate^1–4^. Despite impressive advances in melanoms's surgical therapy, immunotherapy, gene therapy, and more, according to the American Cancer Center, about half of the melanoma patients develop drug resistance in long-term treatment or have a poor prognosis due to late diagnosis^5–9^. Therefore, sensitive methods need to be proposed to evaluate the clinical data of melanoma patients to promote the development of personalized medicine.

Toll-like receptors (TLRs) are a class of transmembrane pattern recognition receptors (PRRs) that are essential components of the innate immune system^10^. The TLR protein family is all glycoproteins, with the N-terminus heading towards the Leucine-rich repeat (LRR) domain with substrate binding ability outside the cell, and the Toll/interleukin-1 (IL-1) receptor homology (TIR) domain responsible for transposing signals into the cell^11^. TLRs have the potential to recognize a diverse array of ligands, including pathogen-associated molecular patterns (PAMPs) and damage-associated molecular patterns (DAMPs)^12^. PAMPs are characteristic molecular structures associated with microorganisms, such as bacterial and viral nucleic acids (unmethylated double-stranded DNA (CpG) and single-stranded RNA (ssRNA)), lipoproteins, lipopolysaccharides (LPS), and flagellar proteins. Additionally, TLRs are capable of detecting DAMPs released by damaged or dead cells, such as Calreticulin exposed on the cell surface, high-mobility group proteins (HMGB1) secreted by tumor cells, ATP molecules released by cells, and heat shock proteins (HSP70, HSP90). These diverse ligands activate downstream signaling pathways, resulting in the production of pro-inflammatory cytokines, chemokines, and other mediators of inflammation. The recognition of both PAMPs and DAMPs by TLRs is critical for the activation and coordination of innate immune responses, as well as for the maintenance of tissue homeostasis. The identification of Toll-like receptors (TLRs) as capable of recognizing conserved structural elements of pathogens has significantly contributed to the current understanding of the mechanisms by which the human body detects pathogenic invasions, elicits innate immune responses, and initiates subsequent adaptive immune responses^13^. Furthermore, it has been established that TLR activation also plays a contributory role in the pathogenesis of several human diseases, including atherosclerosis, systemic lupus erythematosus, and various types of cancer^12^.

In recent years, a growing body of evidence has implicated various components of the immune system in the pathogenesis and progression of melanoma. Notably, immune checkpoint inhibitors targeting molecules such as PD-1, PD-L1, and CTLA-4 have emerged as promising therapeutic modalities for the management of melanoma^14–16^. However, these treatments are only effective in a small number of patients, and as cancer progresses, a large number of patients respond poorly to or even become resistant to such therapies, and primary and acquired resistance to immune checkpoint blockade also become common causes of poor prognosis in patients^17^. Previous studies have shown that TLR involvement increases cancer growth and immune escape and induces apoptosis inhibition and chemoresistance in some tumor cells^18^, including lung cancer^19^, breast cancer^20^, and glioma^21^, such conclusions have not been widely revealed in melanoma.

For this purpose, our study selected 102 TLR-related genes and used the TCGA database to analyze the CNV, SNV, and mRNA expression of these genes in different human cancers, as well as the relationship with patients’ prognosis. Then, by using cluster analysis, we divided melanoma patients into three groups according to TLR-related gene expression, and explored the correlation between TLR pathway and melanoma patients' prognosis, immune infiltration, immunotherapy, pathological features, metabolic reprogramming, and oncogene expression characteristics, aiming to accurately interpret the mechanism of TLR pathway in melanoma. We also screened 6 TLR-related genes to establish a prognostic model of melanoma, and for the 6 modeled genes, we finally analyzed their clinical relevance and single-cell sequencing results separately.

## Materials and methods

### Data collection and processing

The Cancer Genome Atlas (TCGA) database, initiated in 2006 by the National Cancer Institute (NCI) and the National Human Genome Institute (NHGRI) under the National Institutes of Health, is a comprehensive repository of genome-wide expression data. This data is generated through extensive gene sequencing and multidimensional analysis, with the primary objective of mapping human cancer genes. The goal is to enhance our comprehension of the fundamental mechanisms driving cancer, ultimately leading to advancements in cancer diagnosis and treatment strategies^22^. The Gene Expression Omnibus (GEO) database serves as a global open-source functional repository, housing data from high-throughput microarrays and next-generation sequencing studies^23^. The present study entailed the acquisition of TLR-associated mRNA expression profiles, single nucleotide variant (SNV) data, copy number variation (CNV) data, as well as clinical data for patients with diverse human cancers, sourced from the TCGA database. The GEO database is also used to obtain transcriptome and clinical features of melanoma patients. 102 genes related to TLR signaling pathways were downloaded from the MSigDB website (http://www.gsea-msigdb.org/gsea/msigdb/cards/KEGG_TOLL_LIKE_RECEPTOR_SIGNALING_PATHWAY)^24–26^.

In addition, a total of 922 samples from melanoma cancer patients were collected, and their prognosis was followed up for more than 30 days. Specifically, TGCA and GEO cohorts provided 448 and 474 melanoma samples, respectively. The “caret” package in R^27^ was utilized to randomly divide 448 melanoma samples from TCGA platform as a ratio of 6:4. Of note, 60% TCGA SKCM samples were considered as the training dataset (cohort 1, n = 272); 40% TCGA SKCM samples were defined as the internal validation set (cohort 2, n = 176); All of TCGA SKCM samples were also defined as the internal validation set (cohort 3, n = 448); 474 samples from the GEO database were used as the external validation set (cohort 4). All data were analyzed after log transformation and de-batch correlation^28,29^. In the process of molecular typing, in order to make the sample size as comprehensive as possible and the typing results more stable, we conducted cluster analysis on 922 melanoma samples. During the establishment of the prognostic model, in order to make the model construction more robust and realistic, we used Cohort 1 as the training set for modelling, while Cohorts 2–3–4 were used as internal and external validation sets to verify the reliability of the model.

### Pan-cancer analysis

In recent years, many studies have been carried out on the relationship between TLR-related genes and cancer. To outline the multi-cancer characterization of TLR, we analyzed mRNA, SNV, and CNV data from 102 TLR-associated genes from the TCGA database in different human cancers, as well as patient survival data, and visualized them in the form of heat maps^30^.

### Cluster analysis based on TLR expression

Given the notable variances in TLR gene expression and variation levels observed through pan-cancer analysis, a classification model was developed to accommodate these disparities in TLR expression levels among distinct melanoma samples. Specifically, ssGSEA analysis was used to assess TLR pathway activity in each patient. "gplots" packages in R Studio are used for variance analysis, and "pheatmap" packages are used to create heat maps based on cluster analysis results. According to the mRNA expression level of TLR-related genes, we classified all samples into TLR active cluster (C1), TLR expression normal cluster (C2), and TLR expression inactive cluster (C3). To effectively illustrate the correlation between the gene expression levels of the three aforementioned clusters, we employed the "ggpubr" package to generate a violin plot visualizing the enrichment scores of said clusters. To investigate the disparity in patient outcomes among the three aforementioned clusters, the "survival" package and "surminer" package within R Studio were utilized.

### Analysis of tumor purity and (anti-) oncogenes among the three clusters

The pathways related to Toll-like receptors (TLRs) have a significant influence on the regulation of tumor cell proliferation, differentiation, and apoptosis. Notably, these pathways affect tumor purity, as well as the expression of both oncogenes and tumor suppressor genes. To further elucidate these effects, the expression levels of several classical oncogenes and tumor suppressor genes across the three identified clusters were evaluated and presented in the form of heat maps. Apart from tumor cells, tumor tissues comprise immune cells, stromal cells, mesenchymal cells, and other non-tumor cells, all of which collectively influence tumorigenesis. The term "tumor purity" denotes the percentage of tumor cells present within the tumor tissue. ESTIMATE analysis is a computational method that enables the estimation of the relative proportions or abundance of immune cells, stromal cells, and tumor cells present within the tumor microenvironment of the tumor tissues. In our study, the box plot was utilized to illustrate the tumor purity, stromal score, immune score, and ESTIMATE score variations among the three identified clusters.

### Analysis of immune infiltrates and immune checkpoint genes among the three clusters

To assess the distinctions in immune microenvironments among the three identified clusters, the "ggplot2" and "dplyr" packages within R Studio were utilized. Spearman correlation coefficient was adopted as the analytical tool for statistical data analysis, and a heat map was plotted to showcase the association between TLR pathways and the levels of immune cell infiltration. In order to determine the discrepancies in the expression of immune checkpoint genes (ICGs) among the three identified clusters, the Kruskal–Wallis (K–W) test was performed. To evaluate and visually represent the association between TLR scores and immune substances, various R Studio packages, such as "ggstatsplot", "data.table", "dplyr", "tidyr", and "ggplot2", were employed. The generated graph depicted the correlation between the variables through the size of each sphere, while the p-value was reflected through color coding. To demonstrate the correlation between six immune cells or functions (CCR, TIL, Check Point, pDCs, HLA, Parainflammation) and TLR scores, a scatter plot was generated through the utilization of the "ggscatterstats" package.

### Establishment of a new prognostic model based on TLR-related genes

To investigate whether TLR-related genes have a prognostic significance, we conducted univariate Cox regression analysis with a filter criterion of *p* < 0.1. To prevent overfitting and identify suitable variables, we used least absolute shrinkage and selection operator (LASSO) regression analysis. We then used multivariate Cox regression analysis to establish a prognostic risk model comprising six genes (CXCL9, MAPK10, LBP, IFNAR2, RAC1, and TLR2). Note, the aforementioned analyses were all conducted based on the training cohort (i.e. Cohort 1).

### Internal and external validation of the prognostic model

For model validation, the samples in cohort 2, cohort 3, and cohort 4 were divided into low-risk and high-risk groups based on the median score: (1) The diagnostic performance of the prognostic model was evaluated using the area under the receiver operating characteristic (ROC) curve (AUC)^31^; (2) The R package "pheatmap" was employed to create a heat map that displays the contrast in gene expression levels of the prognostic model between the high-risk and low-risk groups.; (3) The Kaplan–Meier method was used to perform survival analysis and evaluate the association between the prognostic model and patient survival^32^; (4) The differences in tumor purity, stromal score, immune score, and ESTIMATE score between the high- and low-risk groups were analyzed using box plots; (5) To gain a better understanding of the differences in immune cell infiltration between high-risk and low-risk groups, we utilized various bioinformatics algorithms, including QUANTISEQ, MCPCOUNTER, EPIC, XCELL, CYBERSORT, and TIMER, with the assistance of the TIMER2.0 database^33,34^.

### Clinical correlation analysis and single-cell sequencing analysis of model genes

Finally, based on the "*BEST"* public website platform (https://rookieutopia.com/app_direct/BEST/), we obtained the correlation between 6 model genes and the clinical characteristics of melanoma, including grade, staging, metastasis, and response to immunotherapy, and the results were displayed in the form of box plots. Similarly, the TISCH2 website (http://tisch.comp-genomics.org/) is a large online database focused on single-cell RNA-seq for studying TME heterogeneity across various cell types^35^. We evaluated each model gene in the melanoma single-cell sequencing map with the help of TISCH2 platform^36^.

## Result

### Pan-cancer analysis based on genomics and transcriptomics

**Figure 1:**
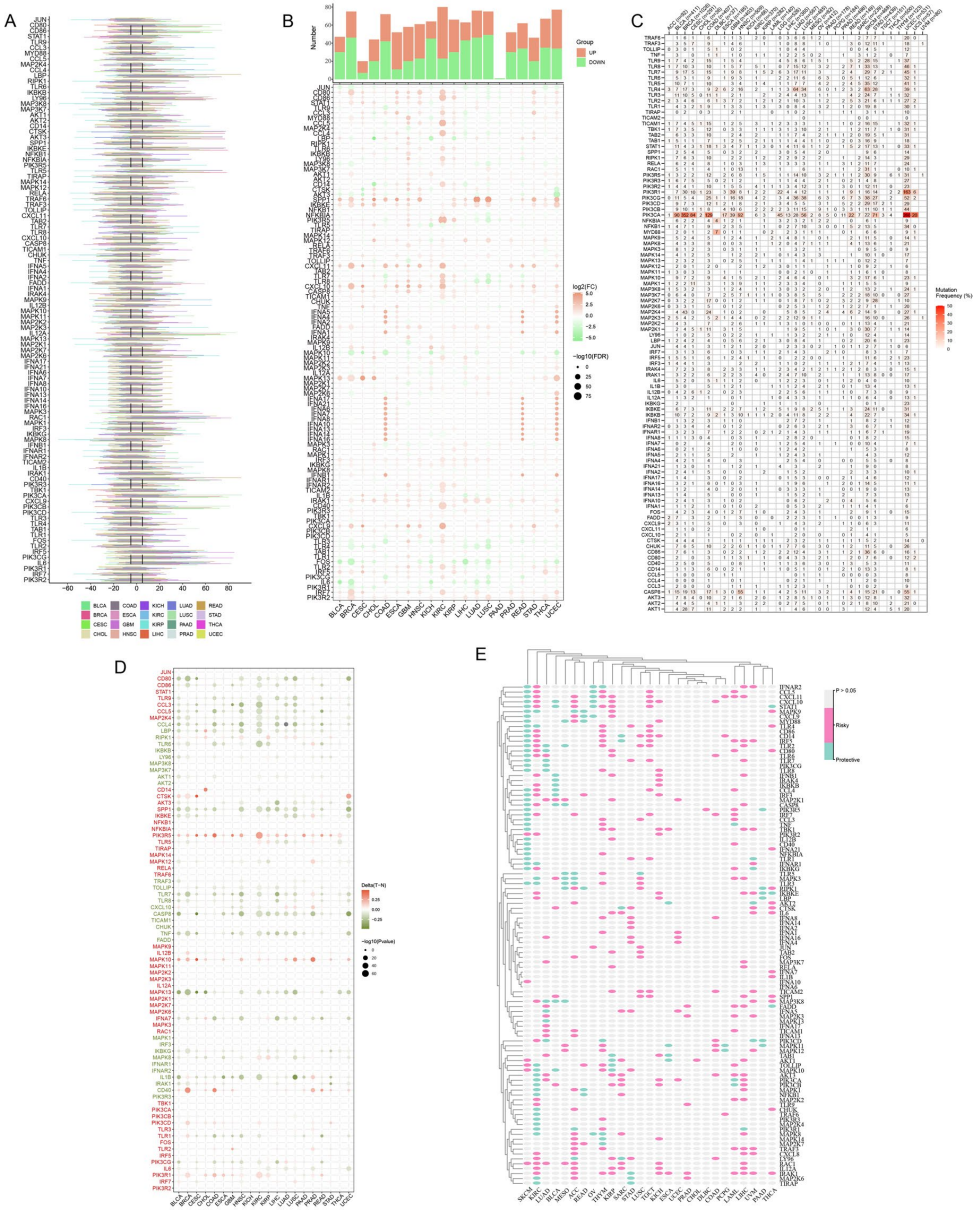
Pan-cancer analysis of TLR-associated genes. (**A**) Data on CNV amplification and loss frequency of TLR-associated genes in different cancer types. The colors of the lines indicate different cancers, and the lengths of the lines indicate the degree of CNV in the genes involved. (**B**) The mRNA expression of TLR-related genes in tumors and adjacent normal tissues was compared, and the red to green circles indicated the expression level from high to low, and the circle size indicated statistical significance. (**C**) SNV frequency data for TLR-related genes in different cancer types, and the numbers indicate the frequency of mutations. (**D**) The degree of methylation of TLR-related genes in various cancers, with the circle color from red to green indicating the degree of methylation of tumor tissue relative to normal tissue, and the circle size indicating statistical significance. (**E**) TLR-related genes are associated with prognosis in various types of cancer, with red and green representing risk genes and protective genes, respectively, and gray indicating non-statistically significant.

To summarize and visualize the changes of 102 TLR-related genes in various cancers, CNV, SNV, and mRNA expression data were visualized as heat maps. CD80, CD86, LY96, CTSK, AKT3, IL12A, RAC1, IRF5, PIK3CG, IL6, and other genes exhibit an extensive expansion of CNV in a variety of cancers. TLR6, MAP3K8, TOLLIP, CHUK, and MAPK8 exhibited extensive deficits (Fig. 1A). In terms of gene expression, SPP1, IKBKE, CXCL11, CXCL10, and other genes showed extensive high expression in COAD, ESCA, KIRC, READ, UCEC, and other cancers. MAPK10, FOS, and IL-6 genes show extensive low expression in LUSC, BRAC, KICH, and other cancers (Fig. 1B). Figure 1C shows the SNV of TLR-related genes, in which the PIK3CA gene exhibits high SNV levels, especially in BRAC, COAD, and UCEC, which may guide subsequent research experiments (Fig. 1C), and the waterfall plot shows different mutation characteristics (Supplementary Fig. S1). In addition, Fig. 1D shows the correlation of TLR-related genes with methylation, prognosis, and cellular pathways in cancer. As shown in the figure, the degree of methylation of PIK3R5, PIK3R1, MAPK10, and CD40 genes in cancer tissues was significantly increased compared to normal tissue, while most of the remaining genes were reduced (Fig. 1D). Finally, in terms of prognosis, TLR-related genes are risk genes for most cancers and protective genes in a few cancers such as melanoma and OV (Fig. 1E).

### Unsupervised hierarchical clustering based on Toll-like receptor pathway scores

**Figure 2:**
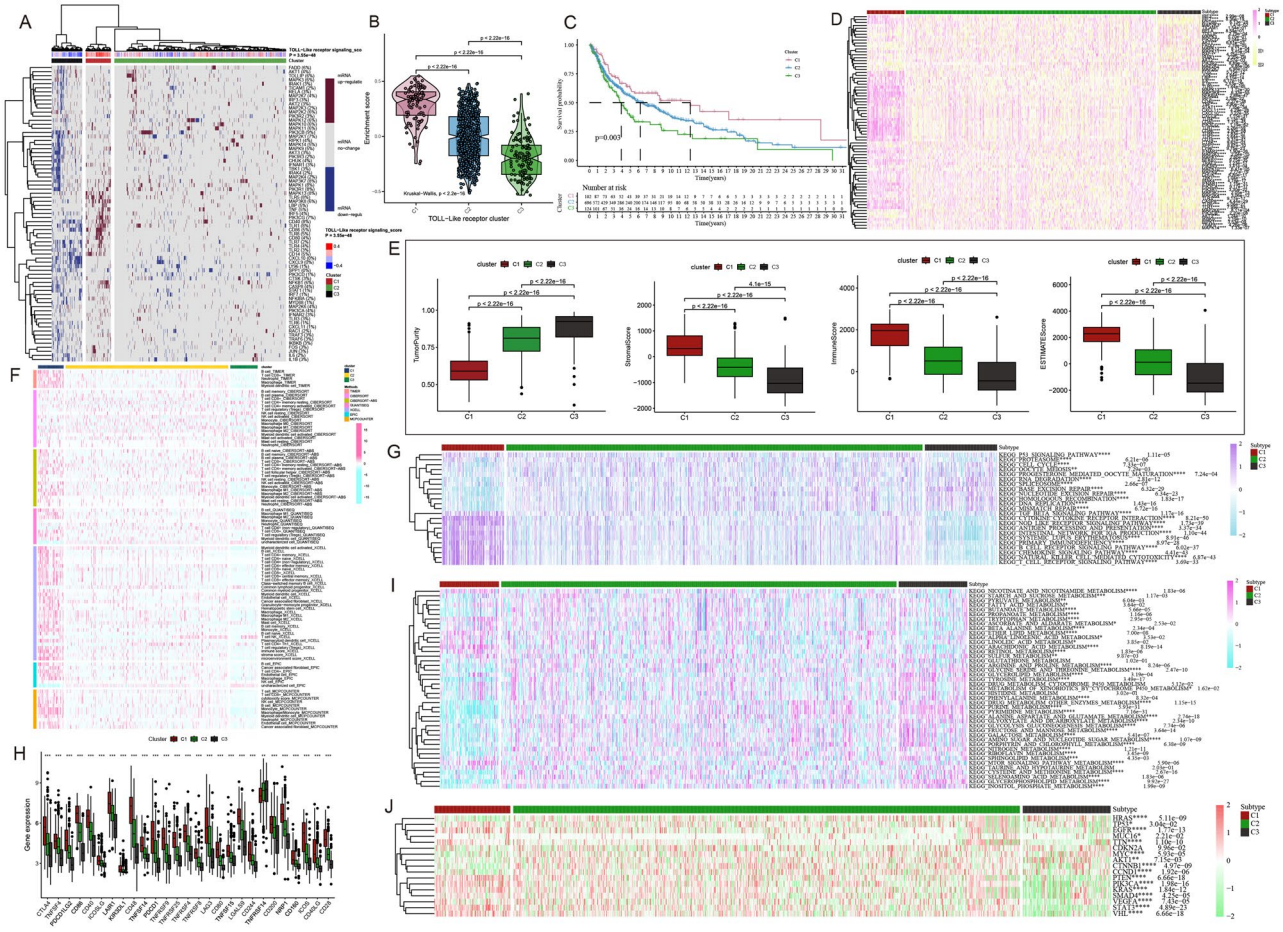
Cluster analysis based on TLR signaling scores. (**A**) Heat map shows that cluster analysis divides all samples into three clusters: active TLR (C1), normal TLR (C2), and inhibited TLR (C3). The red lines in the heat map indicate mRNA up-regulation, the blue lines indicate mRNA down-regulation, and the grey lines indicate mRNA unchanged. The TLR channel score is displayed in four different color bands on the upper end, with red to blue indicating active to suppressed. (**B**) The violin diagram shows that the enrichment fraction of C1 is the highest, C2 is in the middle, C3 is the lowest, and p is at the top of the three clusters. (**C**) Survival curves of the three clusters. C1 has a higher survival rate than C2 and C3. C1 is indicated by a red line, C2 by a blue line, and C3 by a green line. The number of years is expressed as abscissa and survival in ordinates. (**D**) Differences in TLR-related gene expression among the three clusters, pink to yellow show up-regulation to down-regulation. (**E**) The difference among the three clusters in stromal score, tumor purity, immune score, and ESTIMATE score, with p-values shown above. (**F**) Heat map showing differences in immune cell infiltration among the three clusters. (**G**) Differences in the expression of immune pathways between the three clusters. (**H**) Differences in the expression of immune checkpoint-associated genes between the three clusters. (**I**) Differences in metabolic reprogramming between the three clusters. (**J**) Differences in the expression of (anti) oncogenes among the three clusters.

Based on the mRNA expression of TLR-related genes, 922 melanoma patient samples from TCGA and GEO databases were divided into three clusters by the GSVA clustering analysis algorithm, and the TLR signaling pathway score showed different mRNA expression levels (Fig. 2A), and the generated C1, C2, and C3 clusters respectively indicate the active, normal, and inactive TLR signaling pathways. The violin plot shows the degree of difference in TLR-related gene expression between the three clusters, and a *P*-value of < 0.05 indicates that the difference between cluster groups obtained by cluster analysis is statistically significant (Fig. 2B).

The survival curve highlighted the difference in prognosis among patients in the three clusters, indicating that the difference in TLR-related gene expression was related to prognosis, and the results of the violin plot showed that patients with inactive TLR signaling pathway in the C3 cluster had a poor prognosis, and patients in C1 cluster, i.e. TLR signaling pathway turned into active, had a better prognosis (Fig. 2C). The heat map shows the expression of TLR-associated genes in three clusters, with higher expression in the C1 cluster and lower expression in the C3 cluster (Fig. 2D). Figure 2E shows the relationship between the three clusters and stromal score, tumor purity, immune score, and ESTIMATE score, where the C3 cluster had higher tumor purity and lower stromal score, immune score, and ESTIMATE score, suggesting a low degree of immune and stromal cell infiltration, again validating the poor prognosis of patients in the C3 cluster (Fig. 2E). Subsequently, we further analyzed the differences between the three clusters under this cluster analysis in immune cell infiltration (Fig. 2F), immune checkpoints (Fig. 2G), immune-related pathways (Fig. 2H), metabolic reprogramming (Fig. 2I), and (anti-)oncogenes (Fig. 2J), in particular, C1 cluster was obtained with enhanced immune infiltration (B cell, CD8^+^ T cell , Neutrophil, Macrophage, Myeloid dendritic cell, NK cell), up-regulation of immune checkpoint genes(*CTLA4, TNFSF4, PDCD1LG2, CD86, CD40, LAIR,* etc.) and activation of immune pathways, accompanied with mild downregulation of metabolic pathways and active expression of (anti)oncogenes (*TP53, EGFR, PTEN, STAT3,VHL,* etc.). All the results showed that patients in the C3 cluster had poor prognostic characteristics compared with the C1 and C2 clusters.

### Relationship between Toll-like receptors and immune cell infiltration in melanoma

**Figure 3:**
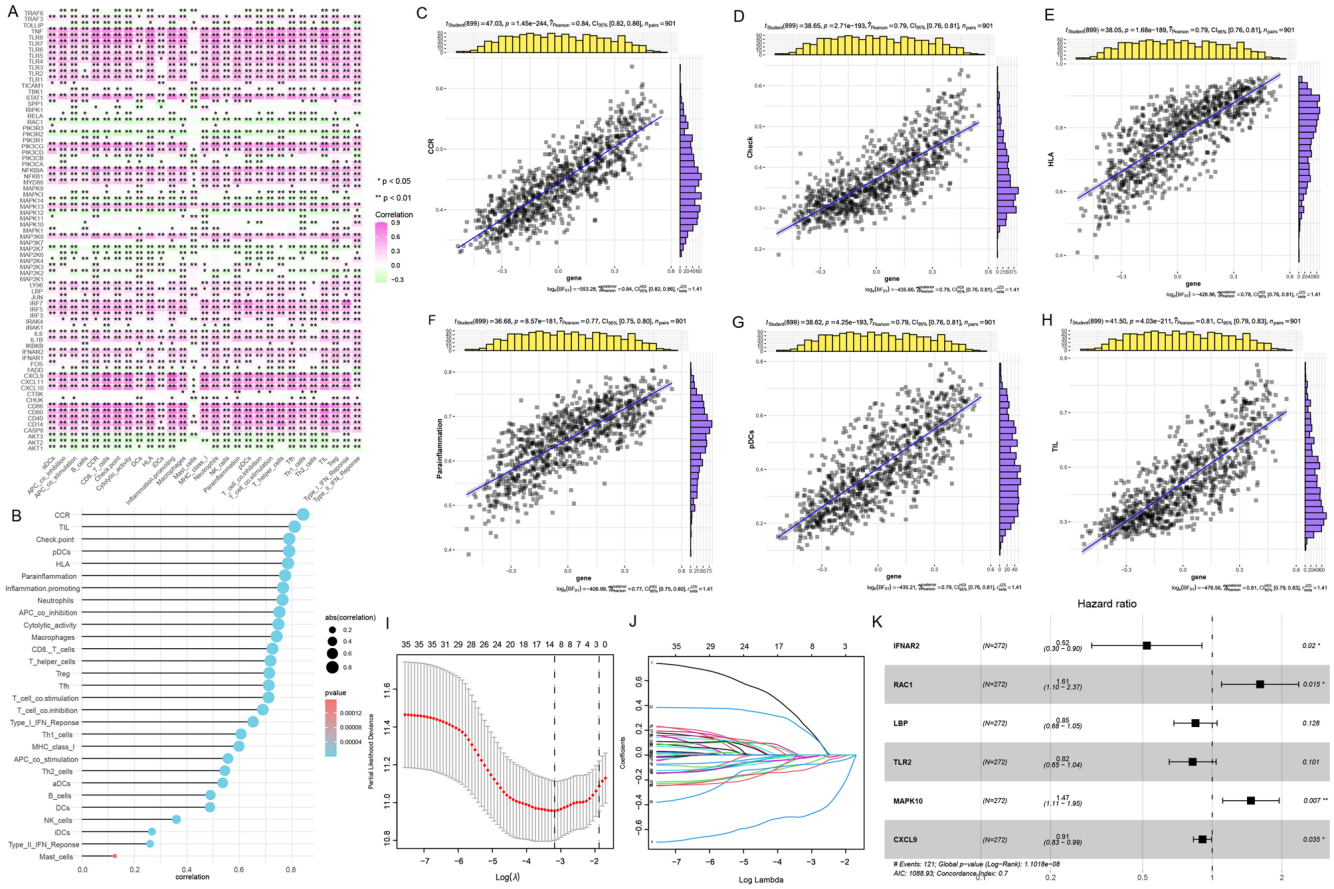
Correlation between TLR pathway score with immune cell infiltration. (**A**) Correlation between TLR-related genes and immunocyte infiltration. (**B**) Correlation between TLR-related scores and immunocyte infiltration. (**C–H**) Scatterplots depict the relationship between TLR scores and six immunoinfiltration-related substances. CCR, Check Point, HLA, Parainflammation, pDCs, TIL were positively correlated. (**I**) LASSO regression determination of candidate genes. (**J**) LASSO coefficient profile of TLR in melanoma. (**K**) Multivariate Cox regression screened out 6 genes to construct a prognostic model.

In recent years, immunotherapy has attracted a lot of attention in cancer treatment, and to test the feasibility of immunotherapy related to the TLR pathway in melanoma, we investigated the relationship between TLR and the immune microenvironment. In this part, we found that TLR-related genes are closely correlated with immune cell infiltration, including both positive correlation (purple) and negative correlation (green), which is related to TLR itself being an important component in the body's innate immunity (Fig. 3A). The bubble chart shows the correlation sequence between TLR score and immune cells and immune function, all of which are positively correlated, and the abs (correlation) is high (Fig. 3B). We then selected the six immune cells or functions with the highest abs (CCR, Check Point, HLA, Parainflammation, pDCs, TIL) were analyzed for correlation with TLR-related genes, and the r values were 0.84, 0.79, 0.79, 0.77, 0.79 and 0.81 respectively, all of which were obvious positive correlations (Fig. 3C–H).

### Establishing a prediction model using LASSO regression

To explore the relationship between TLR pathway and patients' prognoses in melanoma, we divided the clinical data collected from TCGA and GEO databases into four cohorts, which were mentioned earlier, where cohort 1 was used to construct prognostic models and the remaining three cohorts were used for multi-factor validation of models. For the 102 TLR-related genes screened, we first used univariate Cox regression (*p* < 0.1) to screen for survival-related genes, and then used Lasso regression analysis and multivariate Cox regression analysis to obtain the most reliable prognostic markers, and finally obtained 6 genes (*IFNAR2, RAC1, LBP, TLR2, MAPK10, CXCL9*) Composed of a prognostic risk model (Fig. 3I–J). The forest plot shows that *IFNAR2, LBP, CXCL9,* and *TLR2* are the protective genes for melanoma, while *RAC1* and *MAPK10* are the risk genes for melanoma (Fig. 3K). Based on the results of multivariate Cox regression analysis, we finally obtain the calculation scores (i.e. risk scores) of the prognostic risk model. The "predict" algorithm in R was employed to assess the risk scores for each sample.

### Multiple validations of the prognostic model

**Figure 4:**
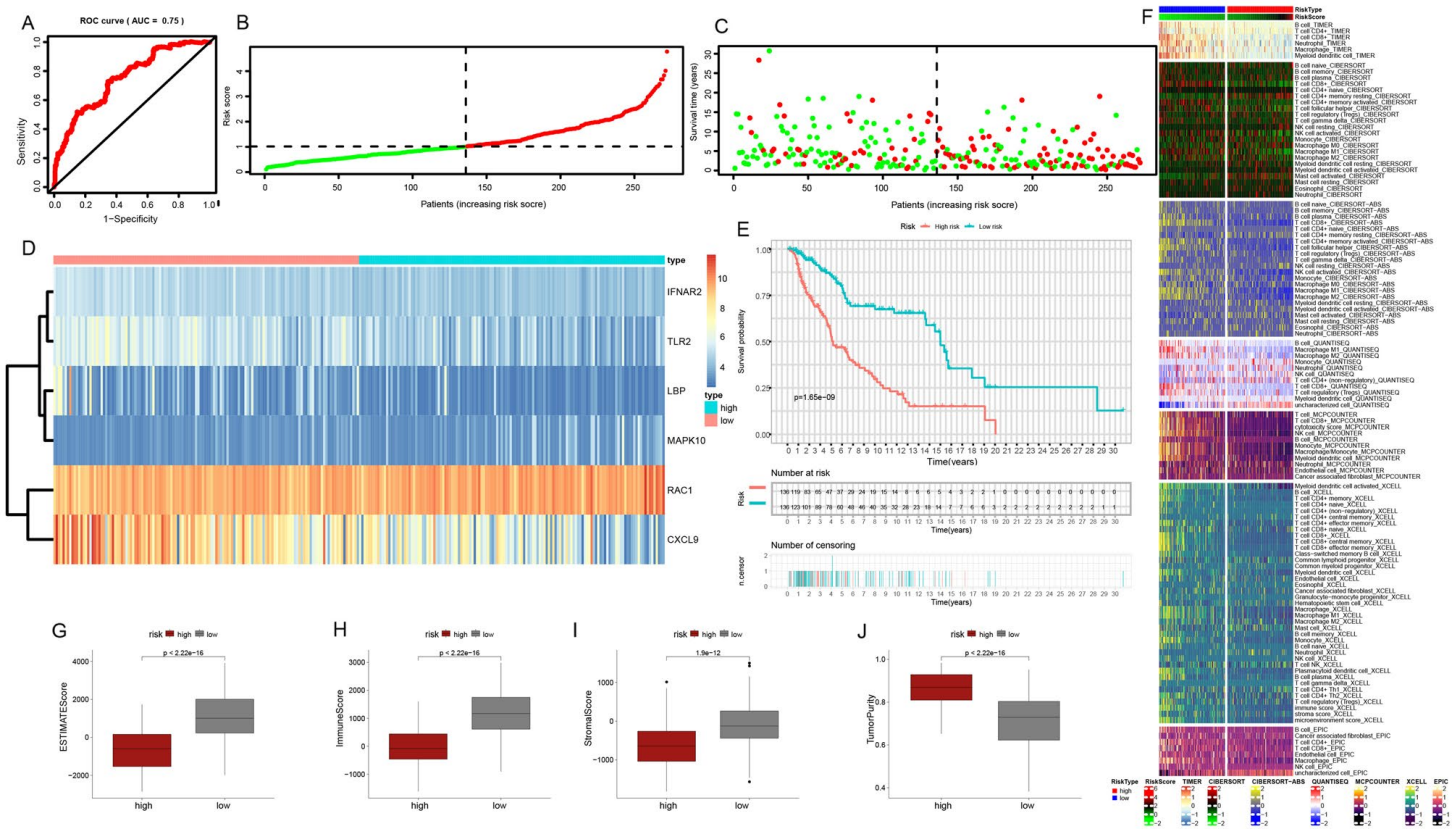
Prognostic model construction in the training set. (**A**) 5-year survival ROC curve for the training set with an AUC value of 0.75. (**B**) Group division. (**C**) Distributions of survival time and status. (**D**) Distributions of model genes expression. (**E**) Prognostic performances of our-established model. (**F**) Assessment of immunocyte infiltration distributions. (**G**–**J**) The difference between ESTIMATE score, immune score, stromal score, tumor purity in the low-risk and high-risk subgroups in the training set.

According to the obtained prognostic risk model, we divided the patients in the four cohorts into a low-risk and high-risk subgroups according to the median prognostic score and further evaluated their clinical value to guide prognosis. In cohort 1, the prognostic performance of the survival model in melanoma patients was analyzed by ROC curve analysis, and the area under the 5-year survival curve (AUC) value was 0.75 (Fig. 4A); Scatterplot showing the relationship between patient risk score and survival (Fig. 4B, C); The survival curve showed that the prognosis of patients in the high-risk subgroup was significantly worse than that of patients in the low-risk subgroup (Fig. 4E). The heat map showed the expression of six model genes in patients in the low-risk and high-risk subgroups, *RAC1* and *MAPK10* expressions were higher in the high-risk subgroup, while *IFNAR2, LBP, CXCL9*, and *TLR2* expressions were higher in the low-risk subgroup (Fig. 4D). Based on various immune cell algorithms, the heatmap indicates that the low-risk group has a higher proportion of immune cell infiltration, suggesting a stronger anti-tumor immune response within the tumor microenvironment of these melanoma patients (Fig. 4F); Fig. 4G–J shows the differences in ESTIMATE score, immune score, stromal score, and tumor purity between low-risk and high-risk subgroups, showing that high-risk groups have higher tumor purity, while ESTIMATE scores are lower.

**Figure 5:**
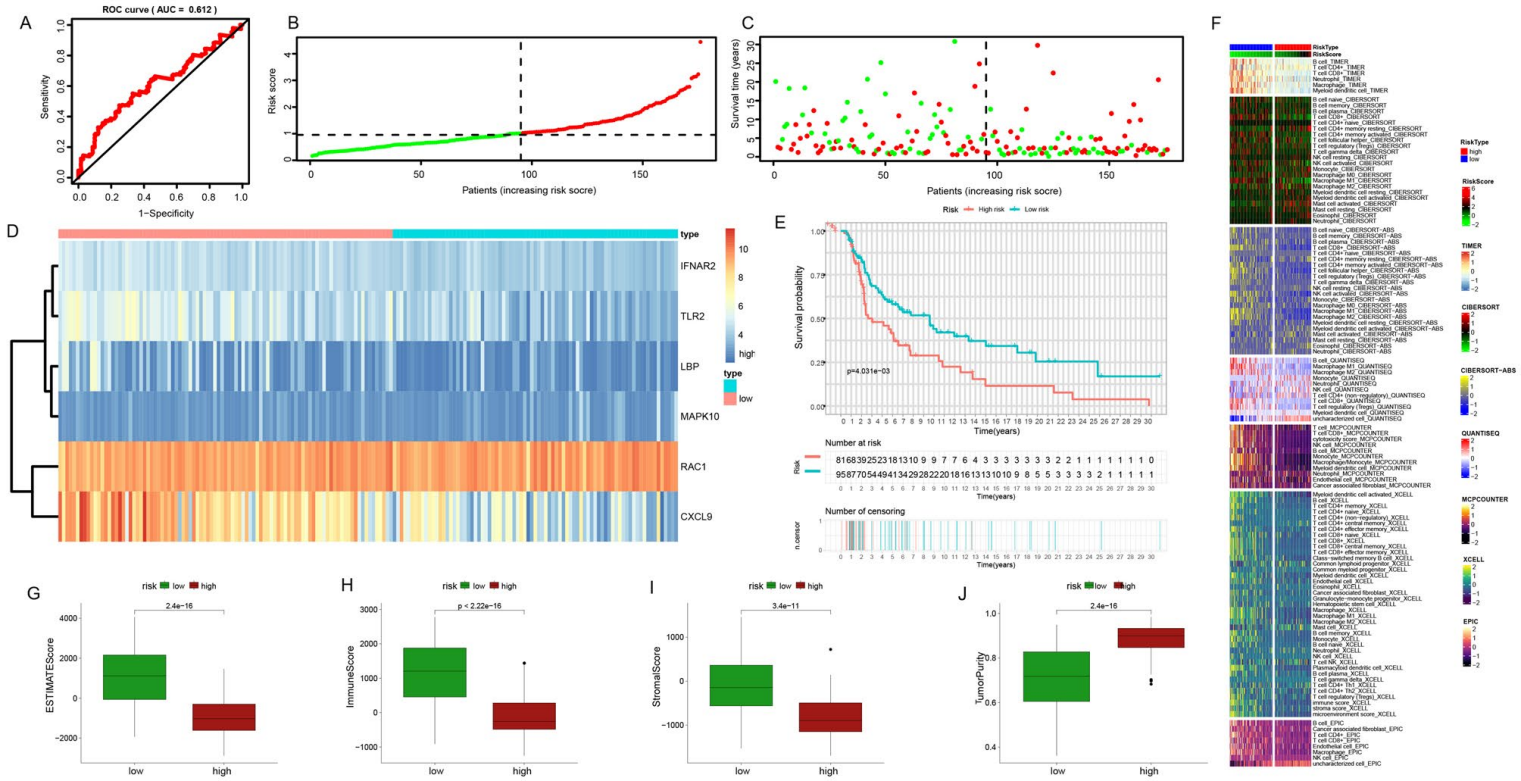
Further validation of the prognostic model in the internal validation set 1. (**A**) 5-year survival ROC curve for the internal validation set with an AUC value of 0.612. (**B**) Group division. (**C**) Distributions of survival time and status. (**D**) Distributions of model genes' expression. (**E**) Prognostic performances of our-established model. (**F**) Assessment of immunocyte infiltration distributions. (**G**–**J**) The differences in ESTIMATE score, immune score, stromal score, tumor purity in the low-risk and high-risk subgroups in the internal validation set 1.

**Figure 6:**
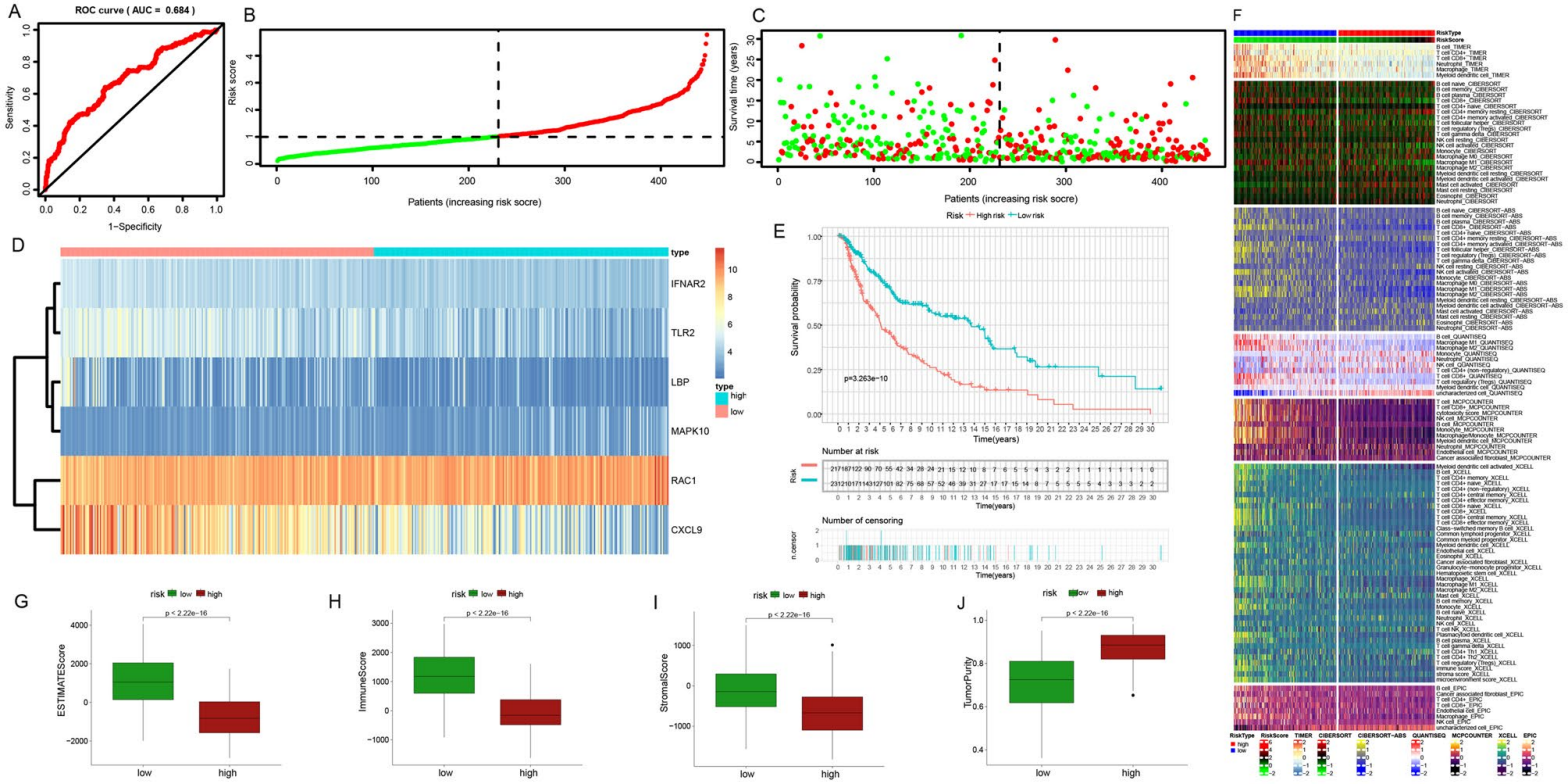
Further validation of the prognostic model in the internal validation set 2. (**A**) 5-year survival ROC curve for an external validation set with an AUC value of 0.684. (**B**) Group division. (**C**) Distributions of survival time and status. (**D**) Distributions of model genes expression. (**E**) Prognostic performances of our-established model. (**F**) Assessment of immunocyte infiltration distributions. (**G**–**J**) The difference between ESTIMATE score, immune score, stromal score, tumor purity in the low-risk and high-risk subgroups in the internal validation set 2.

**Figure 7:**
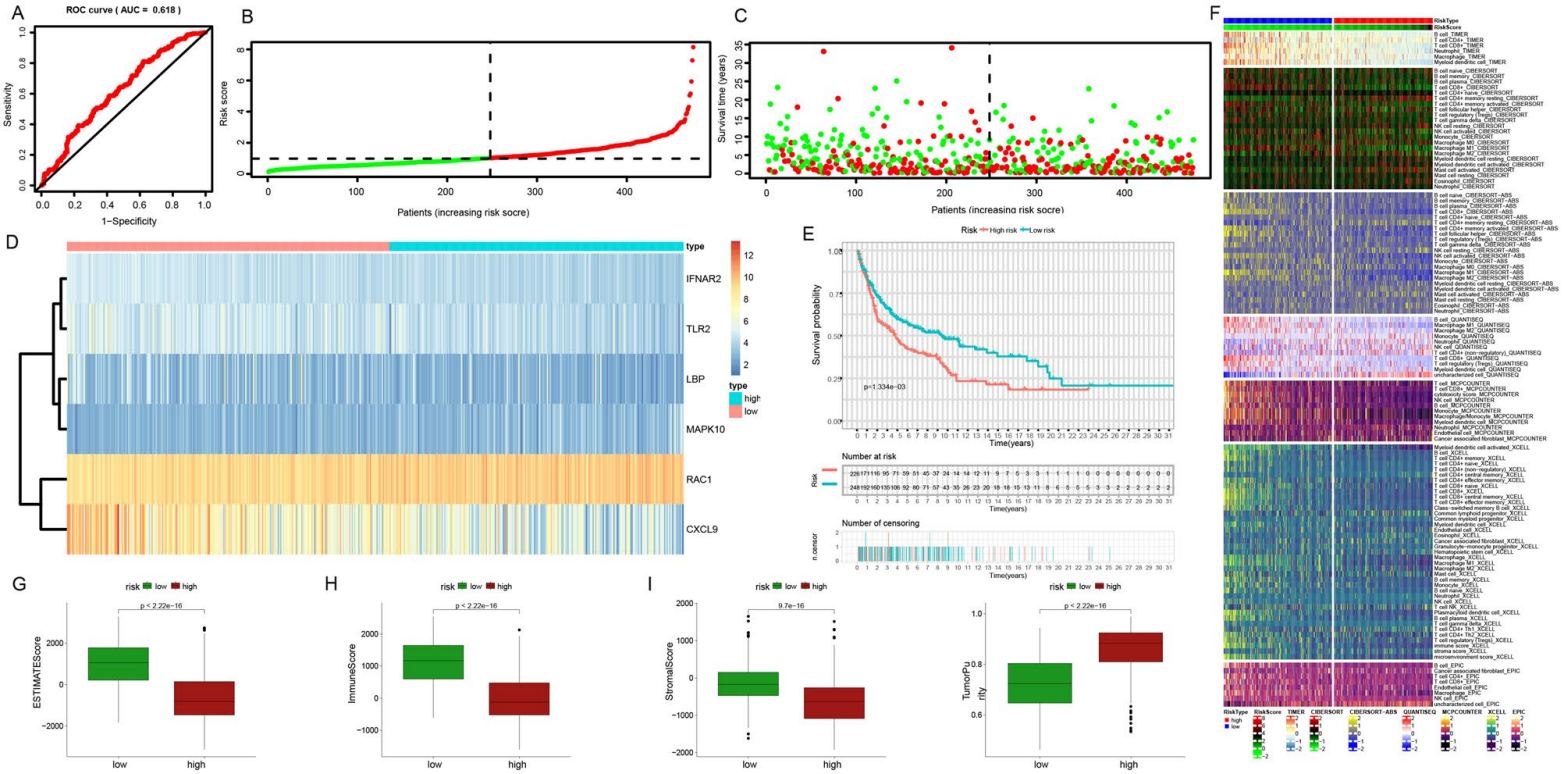
Further validation of the prognostic model in the external validation set. (**A**) 5-year survival ROC curve for the test set with an AUC value of 0.618. (**B**) Group division. (C) Distributions of survival time and status. (**D**) Distributions of model genes expression. (**E**) Prognostic performances of our-established model. (**F**) Assessment of immunocyte infiltration distributions. (**G**–**J**) The difference between ESTIMATE score, immune score, stromal score, tumor purity in the low-risk and high-risk subgroups in the external validation set.

After that, we evaluated the samples in cohort 2 (Fig. 5A–J), cohort 3 (Fig. 6A–J), and cohort 4 (Fig. 7A–J) in the same way. The AUC of the three validation sets was greater than 0.6, and the prognostic differences, tumor purity, immune invasion degree, and gene expression level between the low-risk and high-risk subgroups were the same as those obtained in the training set, that is, the tumor characteristics of the high-risk subgroup samples distinguished by this model were stronger than those of the low-risk subgroup samples. In conclusion, we explore the differences in survival, gene expression, immune function, and tumor purity of different samples from the four cohorts, and verify the accuracy of the constructed risk prognostic model from multiple angles and levels.

### Clinical relevance analysis of the model genes

**Figure 8:**
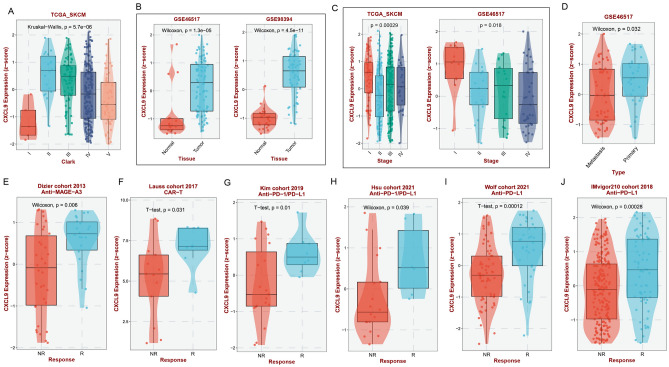
Correlation between model gene CXCL9 with clinical features. Box plots show the relationship between *CXCL9* gene and clark (**A**), cancer tissue expression (**B**), stage (**C**), metastasis (**D**), and immune checkpoint blocker efficacy (**E**–**J**) in melanoma patients, the *p* values are shown above.

To further explore the clinical significance of the six model genes for melanoma, we performed the correlation analysis between the six genes and the clinical characteristics of melanoma, including grade, staging, metastasis, immunotherapy, etc., through the BEST website, and further analyzed the clinical value of the model. Taking the *CXCL9* gene as an example, we found that the expression of the *CXCL9* gene was higher when the Clark grades were II, III, and IV in melanoma patients (Fig. 8A). *CXCL9* gene expression was higher in cancerous tissues compared to normal tissues (Fig. 8B); The expression of the *CXCL9* gene was higher in patients with melanoma when the stage was I (Fig. 8C); Higher expression of the *CXCL9* gene was higher in patients with distant metastases in melanoma (Fig. 8D); In immunotherapy, the expression of *CXCL9* gene was higher in melanoma patients who responded to the immune checkpoint inhibitors (Fig. 8E-J).

 Subsequently, employing a similar approach, we conducted a thorough analysis of the correlation between the clinical characteristics of melanoma and remaining five model genes, i.e. *IFNAR2* (Supplementary Fig. S2A–H), *LBP* (Supplementary Fig. S3A-C), *MAPK10* (Supplementary Fig. S3D–H), *RAC1* (Supplementary Fig. S4A–C), and *TLR2* (Supplementary Fig. S4E–L). In summary, these model genes showed distinct expression levels between tumor and normal tissues. More importantly, these model genes were closely associated with tumor stage, Clark grade, tumor metastasis, immunotherapy response, and etc.  It is precisely due to the significant roles of these model genes in melanoma that further underscores the clinical utility of the prognostic model we have constructed.

### Single-cell analysis of melanoma for modeling genes

**Figure 9:**
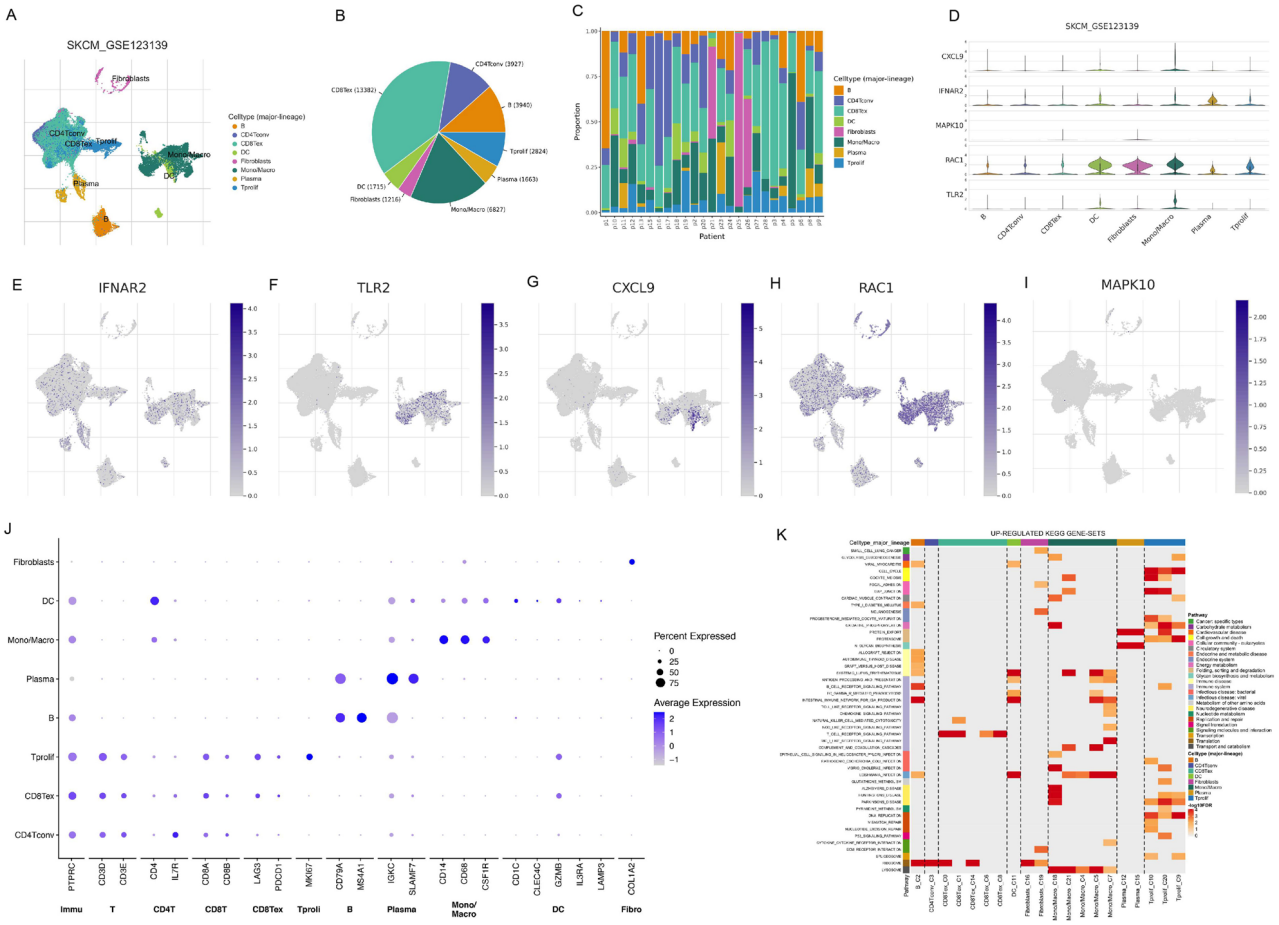
Expression of TLR-related genes in cell subsets in TME. (**A**) Single-cell sequencing identified melanoma cells into 8 subpopulations (B cells, CD4 + T cells, CD8 + T cells, dendritic cells, fibroblasts, monocytes/macrophages, plasma cells, proliferating T cells). (**B**) Number and proportion of each cell subpopulation. (**C**) Distribution of the number of each cell subpopulation in different patient samples. (**D**) Expression of five model genes in different cell subpopulations. (**E**–**I**) Enrichment of five model genes in single-cell sequencing results. (**J**) Maker gene expression for each cell subpopulation, with larger circles or darker colors indicating higher expression. (**K**) Different signaling pathways are enriched into different cell subsets, with the left color block indicating the signaling pathway, the upper color block indicating the cell subset, and the darker the color of the middle part indicates the higher the statistical significance.

Finally, based on the Tisch2 website (http://tisch.comp-genomics.org/home/), we selected the GSE123139 dataset for single-cell analysis to locate genes in the model at the single-cell level to study their potential functions. According to the results of single-cell sequencing, melanoma cell subsets can be divided into 8 types, namely B cells, CD4^+^ T cells, CD8^+^ T cells, dendritic cells, fibroblasts, monocytes/macrophages, plasma cells, proliferating T cells (Fig. 9A). The proportion of each cell type is illustrated in Fig.9B and 9C. For the model genes, our results showed that they were all enriched in these cells (Fig. 9D), where *IFNAR2* is enriched in plasma cells (Fig. 9E), *TLR2* is enriched in monocytes/macrophages (Fig. 9F), *CXCL9* is enriched in dendritic cells and monocytes/macrophages (Fig. 9G), *RAC1* is enriched in fibroblasts, monocytes/macrophages, and dendritic cells (Fig. 9H), and *MAPK10* is enriched in CD8 + T cells and fibroblasts (Fig. 9I). In addition, each cell subset has its marker genes, *CD79A* and *MS4A1* for B cells, *IL7-R* for CD4^+^ T cells, *PTPRC* for CD8^+^ T cells, *CD4* for dendritic cells, *COL12A* for fibroblasts, *CD14* and *CD68* for monocytes/macrophages, *IGKC* and *SLAMF7* for plasma cells, and *MK167* for proliferating T cells (Fig. 9J). Figure 9K shows the relationship between activation of various signaling pathways and single-cell subtypes, in which the TLR signaling pathway is enriched to the monocyte/macrophage system (Fig. 9K), suggesting that the interaction of the TLR signaling pathway with monocyte/macrophages in the immune microenvironment of melanoma may become a research direction for future research.

## Discussion

Melanoma is a highly invasive cancer, so the prognosis of melanoma patients is often closely related to the degree of distant metastasis, and patients with three or more metastases often die within a year, which is a growing concern^37^. Although multiple treatments have been proposed to improve outcomes in patients with melanoma, survival is still limited for most patients with metastatic melanoma^38^. Fortunately, many studies have shown that TLR, as an important member of nonspecific immunity, plays an important role in cancer biology and has the potential to be a prognostic feature of tumor progression, and the immune component in melanoma tissue has also been shown to be useful in assessing the treatment effect and prognosis of patients^39^. TLR agonists have been reported to inhibit melanoma cell growth by inducing DC maturation and T cytotoxicity^22^. To further explore its molecular mechanism, this study explored the specific role of TLR-related pathways involved in melanoma disease progression from multiple angles and levels by including samples from TCGA and GEO databases.

TLRs are transmembrane proteins that recognize molecules from invading pathogens and are important for innate immune and inflammatory responses. The interaction of LRR with PAMPs or DAMPs promotes TIR binding to intracytoplasmic proteins, propagating signals in cells, activating downstream networks such as NF-κB, c-Jun N-terminal kinase, and p38 MAP kinase pathways, further regulating the expression of cancer-related apoptosis and proliferation-related genes^40–42^. The expression characteristics, prognostic value, methylation level and mutation spectrum of TLR-related genes in various human cancers such as BLCA, BRCA, CESC, CHOL, and COAD are shown in the heat map. It can be seen that TLR-related genes have different degrees of mutation and expression differences in a variety of cancers, and bring significant prognostic risks and molecular pathways, and different TLR genes may play synergistic or antagonistic roles in different cancers, which opens up a variety of possible exploration angles for future TLRs in human cancer research.

Furthermore, Toll-like receptors (TLRs) exhibit widespread expression in both tumor cells and tumor-infiltrating immune cells, where they play an intricate role in the regulation of tumorigenesis and anti-tumor immune responses. There is growing evidence that TLR signaling interacts directly with tumor cells or tumor-infiltrating immune cells, participating in important processes such as the activation of immune cells and metabolic reprogramming of tumor cells^43^. Within the tumor microenvironment (TME), Toll-like receptors (TLRs) are expressed in both immune cells and tumor cells, where they play a dualistic role in regulating anti-tumor and pro-tumor responses^21,44^. It has been reported that TLRs expressed on the surface of macrophages activate CD8 + T cells by enhancing the secretion of cytokine IL-12, which may contribute to the regression of melanoma tumors^45^. Therefore, we further divided melanoma patients into three clusters according to the expression level of TLR gene to further explore the specific mechanism. According to the survival curve, the survival status of patients belonging to the C3 cluster (i.e., the group with lower TLR expression) was significantly worse than that of the other two groups, indicating that TLR-related genes were mostly protective. After that, the tumor purity and immune scores of the three clusters were calculated by ESTIMATE analysis, and it was found that patients with low TLR expression had up-regulation of tumor purity and reduction of immune cell infiltration(including B cell, CD8^+^ T cell, Neutrophil, Macrophage, Myeloid dendritic cell, NK cell, etc.), which often results in dysregulation of the tumor's immune microenvironment and poor prognosis. Similarly, similar trends in the activity of immune checkpoint-related genes and classical metabolic pathways have been drawn to previous studies, and our results tend to favor the protective role of TLRs in immune infiltration. Immunocheckpoint inhibitors (ICIs) are emerging as the most effective immunotherapy for cancer treatment. ICI is designed to reverse immunosuppressive tumor environments by targeting immune checkpoint genes (ICGs), thereby enhancing anti-tumor immune responses by activating immunoinfiltrating cells^46–48^. We explored the level of ICGs between three clusters, in which C1 cluster expression was high and C3 cluster expression was low. Metabolic aberrations are prominent features of cancer, and the dysregulated metabolism of cancer cells, characterized by increased aerobic glycolysis and anabolic pathways, plays a pivotal role in various aspects of tumorigenesis, metastasis, drug resistance, and maintenance of cancer stem cells^49^. The high level of metabolic pathway in C3 cluster further suggests the poor prognosis caused by high expression of TLR. In addition, tumor-infiltrating immune cells are an important part of human malignancies, and we also analyzed the correlation between several classical immune cells and TLR scores, including CCR, TIL, CheckPoint, pDCs, HLA, and Parainflammation, the results are all positively correlated. The above results will provide guiding recommendations for immunotherapy in melanoma patients.

Finally, we establish a risk model by LASSO regression analysis to predict the prognosis of melanoma patients. The model was composed of six genes, including *IFNAR2, RAC1, LBP, TLR2, MAPK10, CXCL9*, and multivariate Cox regression analysis showed that *IFNAR2, LBP, CXCL9,* and *TLR2* were the prognostic protection genes of melanoma, while *RAC1* and *MAPK10* were the prognostic risk genes of melanoma, which was consistent with the previous conclusions. For example, interferon α (IFNα) is widely used in the treatment of patients with malignant melanoma, and the interferon α receptor is composed of the IFNAR1 subunit and the IFNAR2 subunit, in which the *IFNAR2* gene plays an important role^50^. *LBP* is mainly expressed in hepatocytes, producing lipopolysaccharide-binding protein, which plays an important role in innate immunity, and its relationship with melanoma has not been explored, and its position in osteosarcoma, lung cancer and breast cancer has been marked as protective. Activation and expression of *CXCL9* gene recruits immune-infiltrating cells such as lymphocytes to TME, thereby enhancing melanoma anti-tumor immunity^51^. The expression of *TLR2* marks the activation of immunity, and L-pampo, as a TLR2 agonist, induces apoptosis and immunogenic tumor cell death by mediating the activation of Th1 and Th2^52^. *RAC1* protein is an Rho GTPase, which can regulate key processes such as melanoma occurrence and metastasis, and over activated *RAC1* signaling pathway induces ERK phosphorylation and PI3K-AKT pathway regulation of melanocytes, promoting the occurrence, progression and metastatic spread of melanoma^53^. The specific mechanism of *MAPK10* acting on melanoma has not been proven, and the carcinogenic effect of *MAPK10* in hepatocellular carcinoma, renal cell carcinoma and gastric cancer has been extensively studied^54–56^.

To verify the accuracy of the model, we divided the sample into four cohorts, and each cohort divided the sample into high and low-risk groups according to the median calculated by the prognostic model formula, and carried out internal validation, external validation, and final test respectively after the model construction was completed, and the results showed that the model had excellent prognosis prediction ability. For model genes, we explored their correlation with the clinical features of melanoma, including grade, staging, metastasis, immunotherapy, etc., to further verify the value and accuracy of the model, this part of the work will be worth in-depth in the future because of the immune regulatory function of TLR-related genes. Finally, using the results of single-cell sequencing, each model gene can be localized to one or several cell types in TME, and we believe that targeting the corresponding gene in the corresponding cell type may be beneficial to manipulate specific cellular components in TME, but the detailed mechanism and feasibility need to be further studied.

In addition, our study had certain limitations. Firstly, this study only includes bioinformatics analysis, and the detailed mechanism of action between TLRs and melanoma requires further validation from in vivo and in vitro experiments to complete our results. Secondly, melanoma is cancer with high metastatic potential, and we did not add an analysis of metastatic sites (bone, lung, liver, and brain), making this experiment incomplete, and future studies need to include a larger number of samples to support our hypothesis.

## Conclusion

By integrating a series of bioinformatics methods, our study identified 6 TLR-related genes (*IFNAR2, RAC1, LBP, TLR2, MAPK10, CXCL9*), which have the potential to serve as new indicators of melanoma progression and prognosis. In addition, based on the TLR score, we divided melanoma patients into three groups, namely the active group, normal group, and inactive group of TLR signaling pathway, and there were significant differences in prognosis, immune invasion, and tumor purity between the three groups, suggesting that the pathogenesis of melanoma is related to abnormal inactive TLR pathway. In conclusion, we created and validated a novel prognostic risk model with good predictive power based on TLR-related genes, which may provide a new strategy with more targeted accuracy for the treatment and prognosis of melanoma patients.

### Supplementary Information

Supplementary Information.

## Data Availability

The datasets analyzed in this work may be found in the Supplementary Materials or contact with the corresponding author (Qihang Yuan, qihangdy@163.com).
