# Supplementary material for: Molecular characteristics and therapeutic implications of Toll-like receptor signaling pathway in melanoma
Source: Sci Rep. 2023 Sep 4;13:13788. doi: 10.1038/s41598-023-38850-y (PMC10477197; doi:10.1038/s41598-023-38850-y)
Supplement: Supplementary file 1 — Supplementary Information. [file 41598_2023_38850_MOESM1_ESM.pdf]

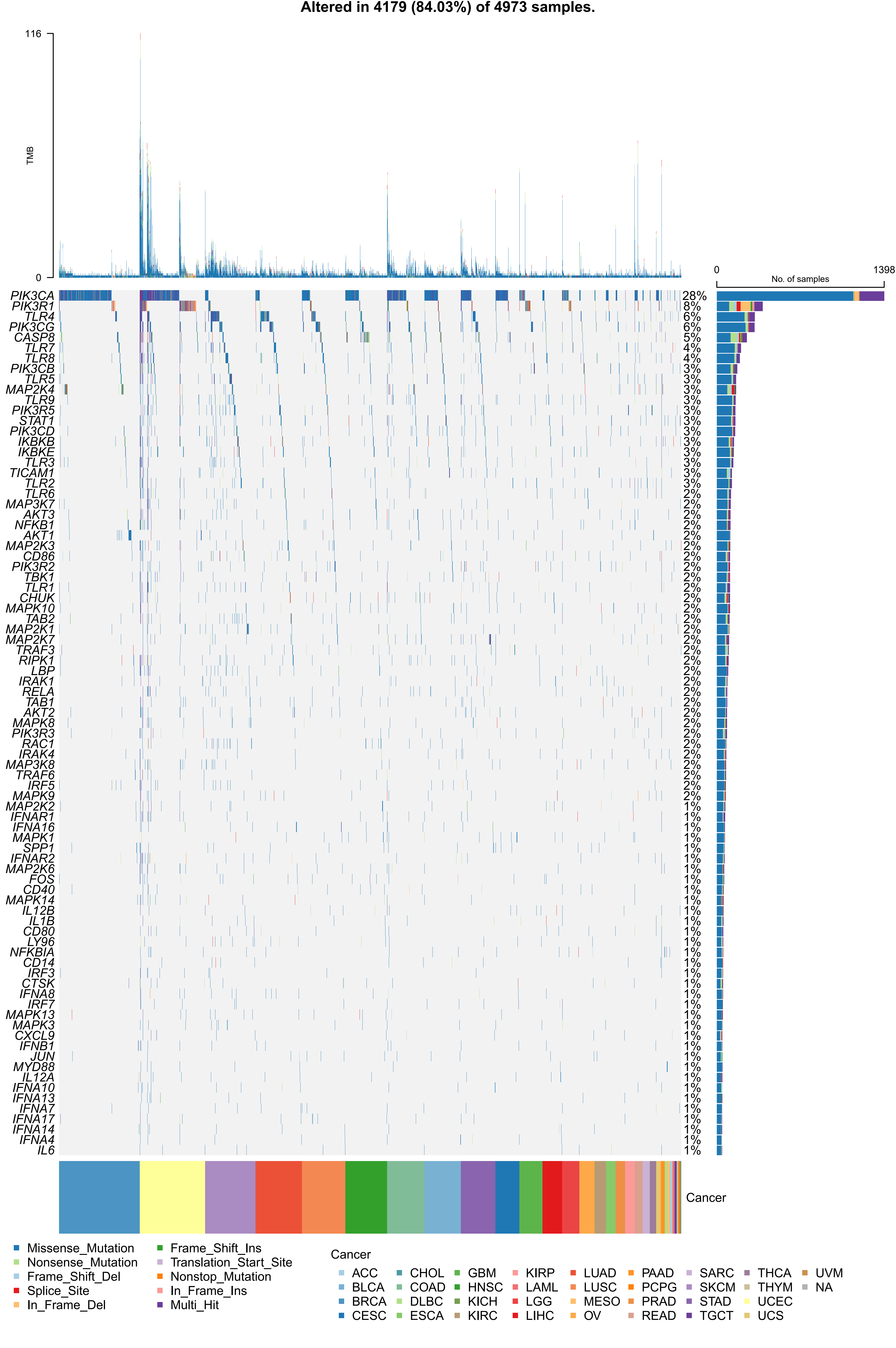

Figure S1 The waterfall plot shows the SNV levels of TLR-related genes in different human cancers, with different colors indicating different mutation characteristics.

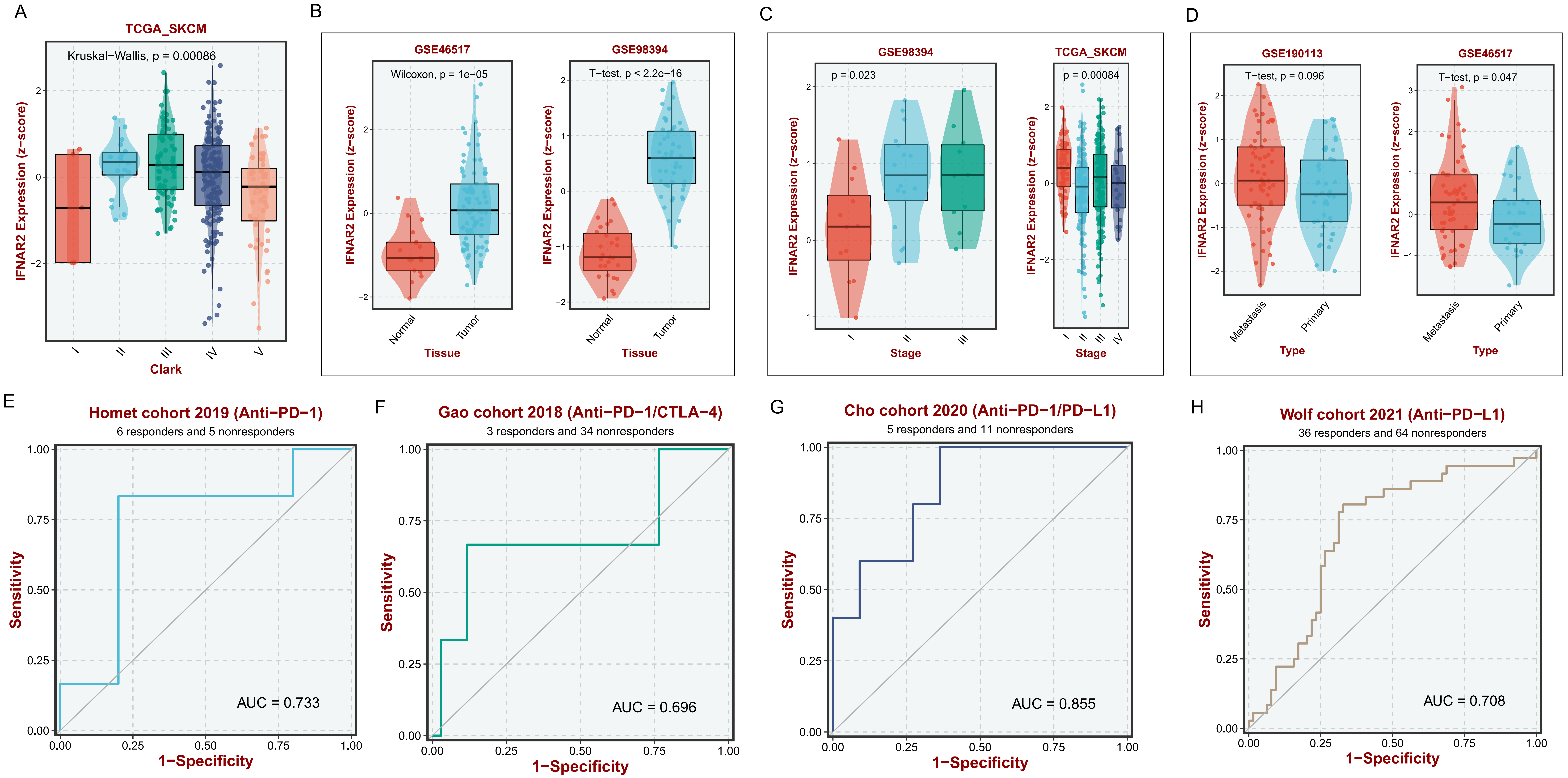

Figure S2 Correlation between model gene IFNAR2 with clinical features. Box plots show the relationship between IFNAR2 gene and clark (A), cancer tissue expression (B), stage (C), metastasis (D), and immune checkpoint blocker efficacy (E-H) in SKCM patients, the p values are shown above.

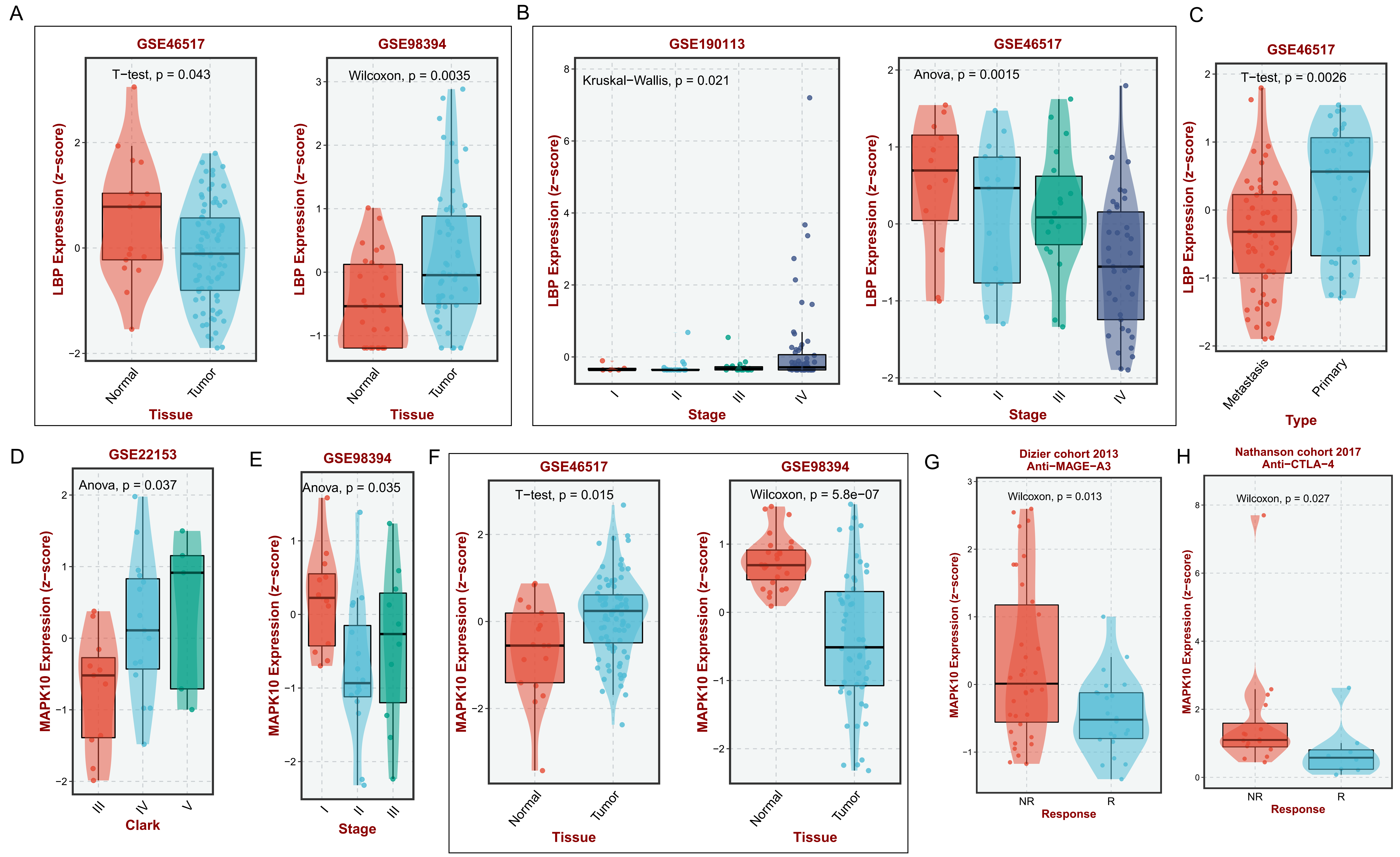

Figure S3 Correlation between model genes LBP and MAPK10 with clinical features. Box plots show the relationship between LBP gene and cancer tissue expression (A), stage (B), metastasis (C)in SKCM patients; the relationship between MAPK10 gene and clark (D), stage (E), cancer tissue expression (F)and immune checkpoint blocker efficacy (G,H)in SKCM patients, the p values are shown above.

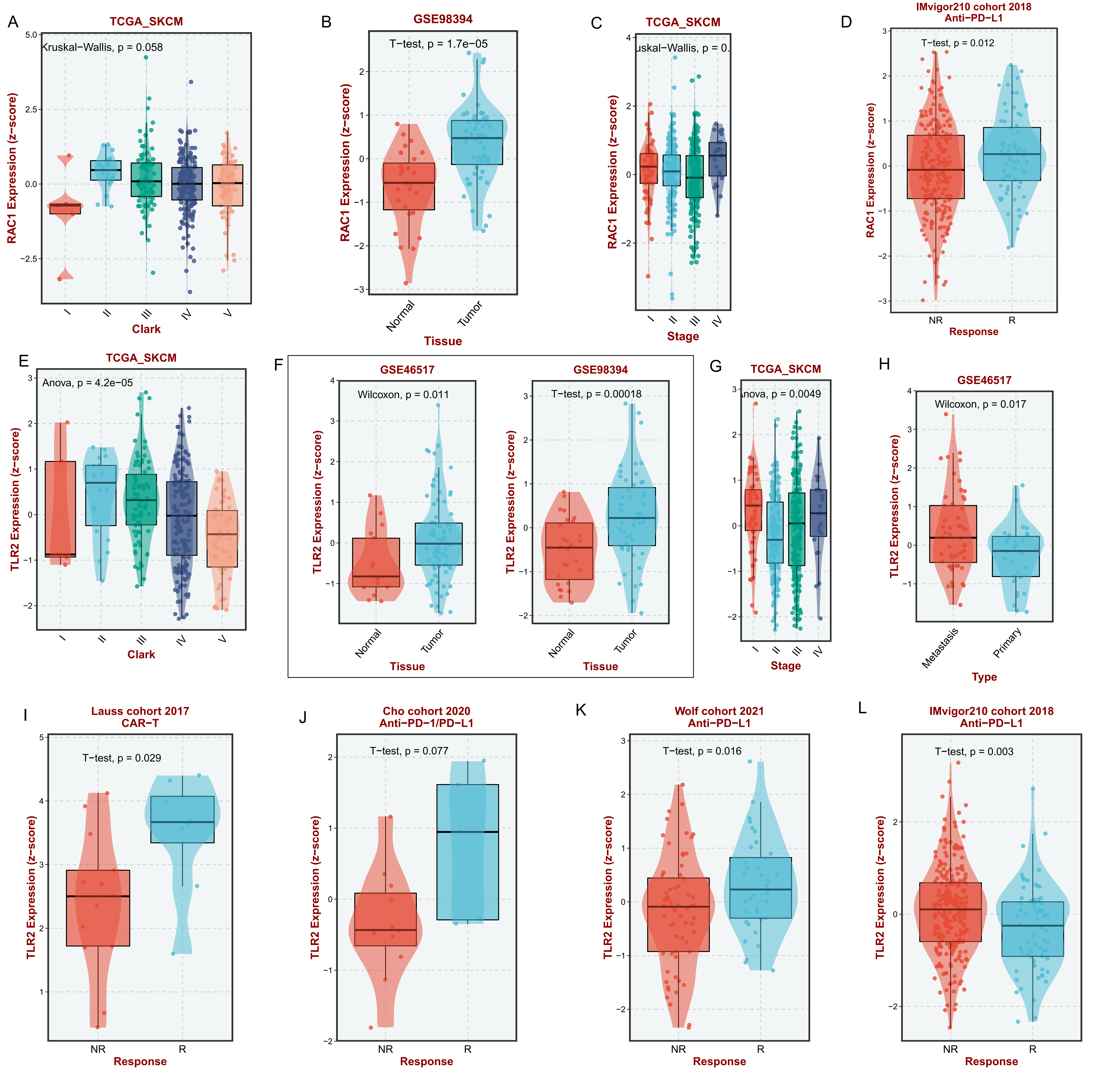

Figure S4 Correlation between model genes RAC1 and TLR2 with clinical features. Box plots show the relationship between RAC1 gene and clark (A), cancer tissue expression (B), stage (C), immune checkpoint blocker efficacy (D)in SKCM patients; the relationship between TLR2 gene and clark (E), cancer tissue expression (F), stage (G), metastasis (H), and immune checkpoint blocker efficacy (I-L) in SKCM patients, the p values are shown above.
